# Physical, chemical and kinetic factors affecting prion infectivity

**DOI:** 10.1080/19336896.2016.1181250

**Published:** 2016-06-09

**Authors:** Francesca Properzi, Anjna Badhan, Steffi Klier, Christian Schmidt, Peter C. Klöhn, Jonathan D. F. Wadsworth, Anthony R. Clarke, Graham S. Jackson, John Collinge

**Affiliations:** MRC Prion Unit, Department of Neurodegenerative Disease, UCL Institute of Neurology, London, UK

**Keywords:** BSE, CJD, cell culture, prion, PrP, RML, scrapie

## Abstract

The mouse-adapted scrapie prion strain RML is one of the most widely used in prion research. The introduction of a cell culture-based assay of RML prions, the scrapie cell assay (SCA) allows more rapid and precise prion titration. A semi-automated version of this assay (ASCA) was applied to explore a range of conditions that might influence the infectivity and properties of RML prions. These include resistance to freeze-thaw procedures; stability to endogenous proteases in brain homogenate despite prolonged exposure to varying temperatures; distribution of infective material between pellet and supernatant after centrifugation, the effect of reducing agents and the influence of detergent additives on the efficiency of infection. Apparent infectivity is increased significantly by interaction with cationic detergents. Importantly, we have also elucidated the relationship between the duration of exposure of cells to RML prions and the transmission of infection. We established that the infection process following contact of cells with RML prions is rapid and followed an exponential time course, implying a single rate-limiting process.

## INTRODUCTION

Mammalian prion diseases are fatal neurodegenerative conditions including kuru, Creutzfeldt-Jakob disease (CJD), Gerstmann-Sträussler-Scheinker syndrome and fatal familial insomnia in humans, scrapie in sheep, bovine spongiform encephalopathy (BSE) in cattle and chronic wasting disease in cervids.^1-4^ They are characterized by the post-translational conversion of host cellular prion protein (PrP^C^) into abnormal disease-related isoforms designated PrP^Sc^.^1-4^ According to the widely accepted ‘protein-only’ hypothesis,^5^ an abnormal PrP isoform is the infectious agent acting to replicate itself with high fidelity by recruiting endogenous PrP^C^ and that the difference between these isoforms lies purely in the monomer conformation and its state of aggregation.^1,2,4,6-10^ However, prion-diseased brain contains a highly heterogeneous population of abnormal PrP isoforms that may differ with respect to their conformation, aggregate size, potential neurotoxicity and specific prion infectivity ^4,11^ and indeed prion strains, rather than constituting a molecular clone, appear to comprise an ensemble or quasispecies maintained under host selection pressure.^4,12,13^ Due to this heterogeneity, the precise structures of the infectious or toxic particles remain unclear.

Much of the effort to elucidate prion structure concentrates on ex-vivo purified material and the mouse-adapted scrapie RML strain,^14^ is used in many laboratories. In the work we describe here, our aim was to make a systematic assessment of the physical stability of RML prions in a range of conditions used in their purification, storage and experimental manipulation as a baseline dataset to aid interpretation of a wide range of experimental work with this prion strain.

This analysis was made possible by the development of the Scrapie Cell Assay (SCA) ^15^ which we have now extended into a semi-automated version (ASCA) that allows us to assess the infectious titer of large numbers of samples with a precision impractical with conventional rodent assays.^11,16^ Neuroblastoma N2a lines have been isolated that are highly susceptible to mouse prions, as shown by propagation of infectivity and accumulation of protease-resistant PrP, so enabling quantitative in-vitro assays for prion infectivity.^11,15-17^ The automated assay allows investigation of many variables in an affordable, rapid and ethical fashion. It also allows exploration of conditions with quantitatively modest, but mechanistically important, effects on infective titer that the imprecision of conventional rodent prion bioassay (with a typical accuracy of around 0.5–1 log LD_50_ ^18^) would simply not allow. Here we report the effects of chemical reduction, detergent treatment, centrifugation, freeze-thawing and contact exposure times on the infectious titer of mouse RML prions.

## RESULTS

### Stability of RML Brain Homogenate Infectivity After Routine Experimental Procedures

#### Freeze/Thawing

**FIGURE 1.**
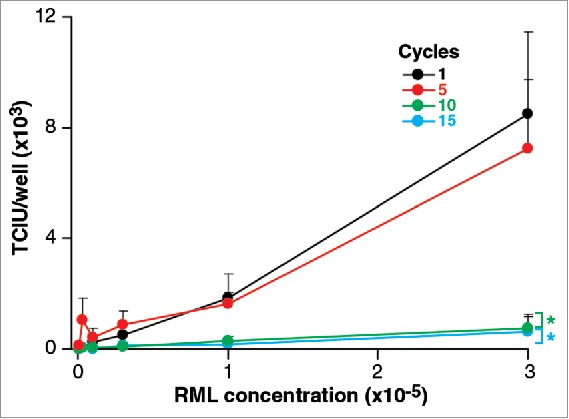
Stability of RML prions after freeeze/thawing. 1 ml of 10% w/v RML brain homogenate (I6200) was repeatedly snap frozen in liquid nitrogen and thawed up to 15 times. Aliquots were removed after 1, 5 10, 15 cycles. Infectivity of RML brain homogenate was quantified with the ASCA as Tissue Culture Infectious Units (TCIU). Statistical analyses were performed using a two tailed t-test by comparison to 1 cycle as a baseline control (n = 6 assay replicates for each cycle; % p < 0.0001).

As shown in Figure 1, aliquots of RML brain homogenate subjected to 5 cycles of freeze/thawing did not show significant changes in infectivity compared to samples frozen immediately. This is an interesting result since it might be argued that disruption as a result of freezing would be likely either to denature the infectious particles or increase their number by fragmentation. The fact that there is no change in the infectivity could imply that neither of these processes occurred or that one compensated for the other. However, after 10 and 15 cycles of freeze-thawing infectivity titres are reduced by about 1 log TCIU suggesting that a denaturing process had become apparent.

#### Incubation at Different Temperatures

**FIGURE 2.**
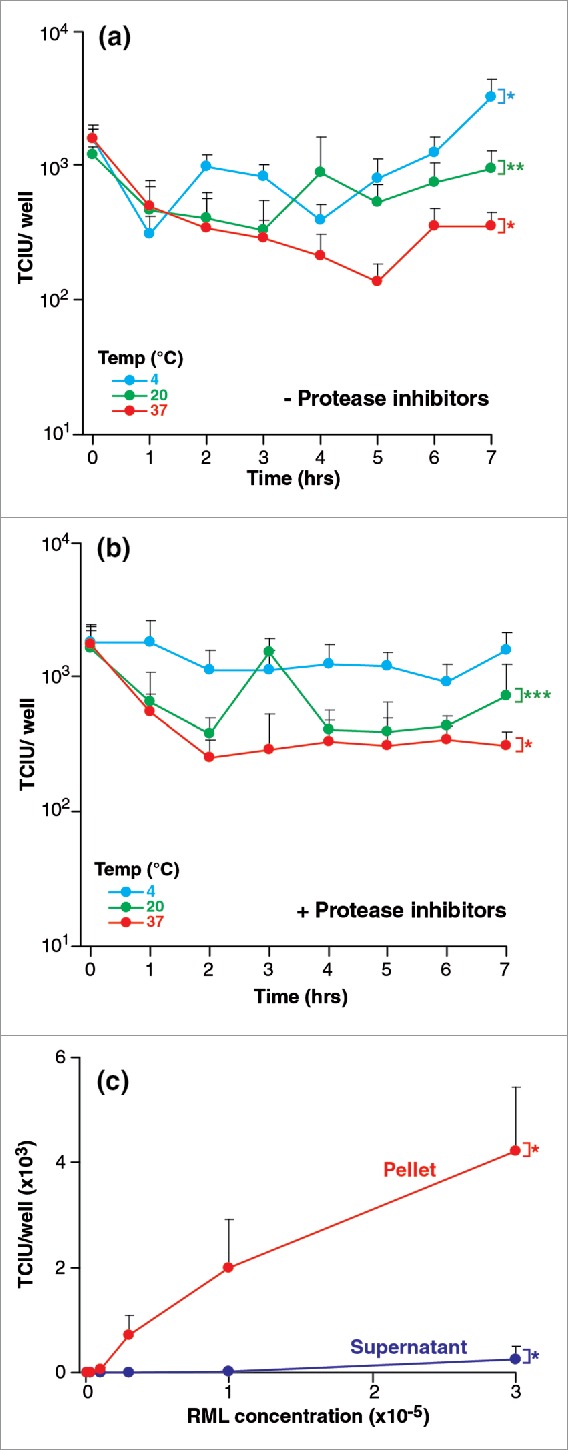
(See next page). Stability of RML prions after various treatments. Infectivity of RML brain homogenate (I6200) was quantified with the ASCA as Tissue Culture Infectious Units (TCIU),. (a,b) Incubation at different temperatures- 10% w/v RML brain homogenate with (a) and without (b) protease inhibitors was incubated without agitation at 4, 20 and 37°C for 7 hours. Aliquots were removed every hour. (c) Centrifugation- RML brain homogenate was centrifuged for 10 min at 13,200 rpm. The pellet was resuspended to the original starting volume and assayed with the supernatant fraction. Statistical analyses were performed using a two tailed t-test (n = 6 assay replicates for each condition). For data in (a) and (b) time = 0 was compared to time = 7 hours (% p < 0.0001; %% p0.0053, %%% p = 0.0045), in panel (c) the partition between pellet and supernatant at the highest concentration of RML was compared (% p < 0.0001).

Despite a 7-hour incubation of homogenate at 4, 20 and 37°C where endogenous proteolytic activity could have a detrimental effect on prion integrity, at worst infectious titer was only slightly reduced by < 1 log TCIU (Fig. 2a). Such losses were not abrogated by inclusion of a commercially formulated mix of protease inhibitors (Fig. 2b).

#### Centrifugation

After 10 min of centrifugation at 13,200 rpm (16,100 × g), the pellet was re-suspended to the original starting volume and infectivity was measured. Only about 2% of the infectious units were in the supernatant (see Fig. 2c) and infectious titers in the re-suspended pellet fraction were the same as those in control samples before centrifugation.

### Reducing Agents; Effects on Infectious Titer, Cell Susceptibility and Cell Viability

**FIGURE 3.**
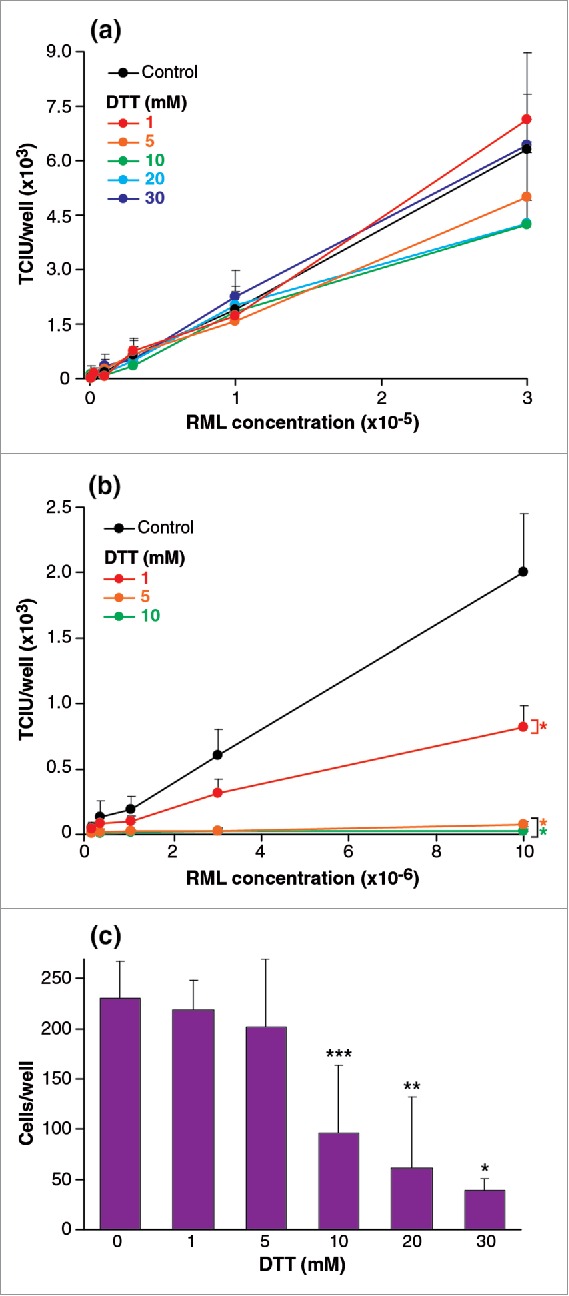
(See next page). Effect of reducing agents on RML brain homogenate infectious titer and on cell susceptibility to prions. (a,b) RML brain homogenate was treated with 1, 5, 10, 20, 30 mM DTT before (a) or after (b) dilution into cell media and ASCA processing. Infectivity was quantified in Tissue Culture Infectious Units (TCIU). In (a) samples were incubated for 1 h before dilution into cell medium (with concomitant dilution of final DTT concentration) and SCA processing. In (b) DTT was added to serially diluted RML during the three infection days. (c) Cell viability was determined by trypan blue staining after three splits of the same ASCA shown in (b). Statistical analyses were performed using a two tailed t-test (n = 6 assay replicates for each condition); in panel (b) comparisons of DTT concentrations to control at the highest concentrations of RML gave; % p < 0.0001. In panel (c) toxicity was significant at 10, 20 and 30 mM DTT relative to the 0 mM control (% p < 0.0001, %% p = 0.0004, %%% p = 0.0018).

RML brain homogenate was treated with 6 concentrations of dithiothreitol (DTT); 0, 1, 5, 10, 20 and 30 mM before being assayed by ASCA. No significant difference was observed in any of the treated samples compared to the controls (Fig. 3a). In contrast, when cells were exposed to a range of DTT concentrations by adding the reducing agent to culture medium during the ASCA incubations, the effects were striking (Fig. 3b). At final concentrations of 1 and 5 mM DTT infectivity was reduced by 2 fold, and 30-fold respectively. The reduction of apparent infectivity was not due to reduced cell viability since final concentrations of 1 mM and 5 mM DTT had no effect on this (Fig. 3c).

### Effects of Lipid Treatments on Infectious Titres of RML Brain Homogenate

**FIGURE 4.**
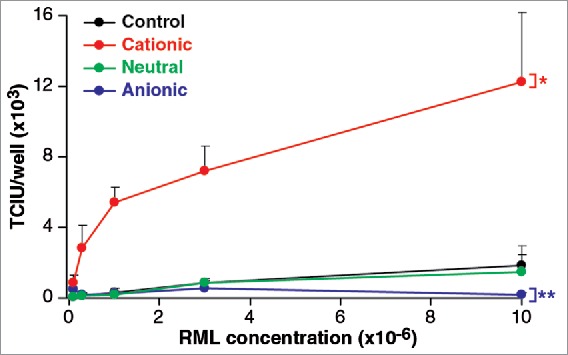
Phospholipid effect on RML prion cell uptake. An anionic, a neutral and a cationic phopspholipid mix were vacuumed dried overnight and separately added to a 0.1% w/v solution of RML brain homogenate. After 5 min incubation and 2 min agitation at room temperature they were serially diluted and processed for the ASCA for the quantification of infectivity in Tissue Culture Infectious Units (TCIU). Statistical analyses were performed using a two tailed t-test comparing the values for various lipids relative to control at the highest concentration of RML (n = 6 assay replicates for each condition) % p < 0.0001, %% p = 0.011).

As it is known that phospholipid molecules influence the uptake of macromolecules into cells, and have been suggested to enhance prion formation and replication ^19-21^ we investigated the effects of three, contrasting lipid mixtures on the efficiency of infection. To examine the effect of anionic, neutral and cationic species, we used a phosphatidyl choline/phosphatidyl glycerol mix, a phosphatidyl choline/phosphatidyl ethanolamine mix and the commercial preparation Pro-Ject mix, respectively. Lipids mixtures were vacuum dried overnight and separately added to a 0.1% w/v solution of RML brain homogenate and sonicated. As shown in Figure 4, the neutral mixture had no effect on the level of infectivity. However, the anionic lipids reduced the infectivity significantly, while the cationic mix had a strong enhancing effect.

### Effect of Cell-Contact Time on the Level of Prion Infectivity

**FIGURE 5.**
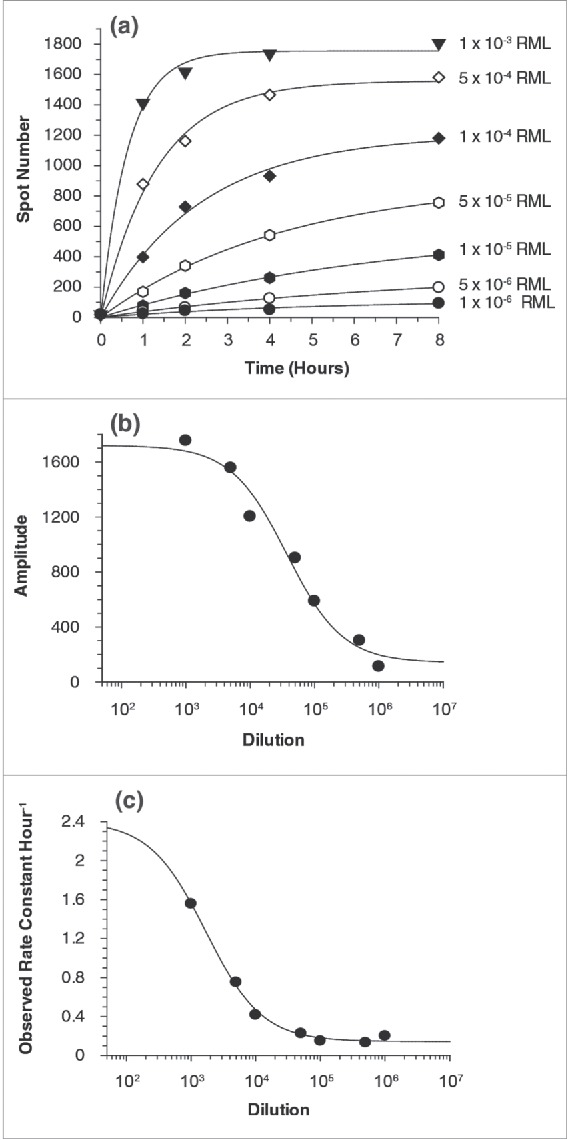
(See next page). Kinetics of RML prion infection. In the standard SCA, cells are incubated with prions for three days before splitting. To assess the kinetic of prion infections the RML incubation period was reduced from 72 to 1, 2, 4 and 8 h. (a) Plot of time course of infection with RML showing the number of spots as a function of the time of exposure at a series of RML prion concentrations. The lines represent fits to the data according to a single-exponential function. (b) Plot of the amplitudes of the fits shown in (a) as a function of the concentration of RML, fitted to a simple hyperbolic binding curve. (c) Plot of the first-order rate constants in (a) as a function of RML concentration and fitted to the equation k_obs_ = offset + k_max_.[RML]/(K+[RML]). The data shown and analyzed are the mean of the two independent data sets.

To examine the kinetics of prion infection, cells were incubated with RML-infected homogenate for different exposure periods before removal by washing and cell passage. Cells were incubated with prions for 72 h, as in the standard scrapie cell assay,^15^ to provide a maximal level of infection and the experiment was repeated twice with exposure times of 1, 2, 4 and 8 hours and the titer was varied from a dilution of 10^6^ to 10^3^ (see Fig. 5a). The time-course of infection reproducibly followed first-order kinetics and could therefore be fitted to an amplitude of effect (Fig. 5b) and a rate constant (Fig. 5c). These reflected the level and the speed of infection, respectively. The fact that the process is exponential with time implies that there is a single rate-determining step in the process of infection and the plot of amplitude versus prion concentration (Fig. 5b) gives an orthodox saturation curve with a pseudo-dissociation constant equivalent to a∼1 × 10^4.6^-fold dilution of the titrated RML sample. Given that the prion titer of the 10% w/v RML brain homogenate is ∼10^7.3^ intracerebral LD_50_ ml^−1^ ^17^ this yields an apparent K_d_ of ∼10^2.7^ infectious units ml^−1^. Interestingly, the kinetics of infection also appear to follow an orthodox mechanism as shown by the asymptotic relationship between prion concentration and the rate of infection of cells (Fig. 5c). This plot shows that the half-maximal rate is reached at a dilution of ∼1 × 10^3.2^-fold, a magnitude 1.4 logs lower than in the equilibrium saturation curve, showing that the initial concentration is 25 times higher. This implies that there is an initial rapid-equilibrium interaction with a component at the cell surface followed by a slower rate limiting event with a rate constant of about 2 hr−^1^ that leads to infection (Fig. 5c), i.e. the slow process occurring after the initial binding step has a half-time of about 20 min.

## DISCUSSION

The speed, throughput and accuracy of the automated scrapie cell assay (ASCA) have allowed us to undertake a detailed evaluation of conditions that influence the apparent infectivity of RML prions. The rationale for this is to derive a better understanding of the characteristics of RML prions, the principal prion strain used experimentally in many laboratories. The ASCA also allows us to explore conditions with quantitatively modest, but mechanistically important effects on infective titer that the relative imprecision of conventional rodent prion bioassay, which typically has an accuracy of around 0.5–1 log LD_50_, precludes. It is also likely that the assay itself might allow detection of infectious units that are, for example, degraded or cleared *in vivo* in rodent assays, as was previously shown in PrP-null mice.^11,22^ In this study our main objective was to quantify the effects of treatments commonly used in prion purification and storage and in the experimental investigation of prion properties.

Repeated rounds of freeze-thawing (> 10) led to a reduction in titer to about 10% of the original. Incubation of homogenates at 37°C in physiological solvent conditions gave a loss of infectious titer <1 log occurring with a half-life of about an hour. The latter was not arrested by the inclusion of proteinase inhibitors in the incubation and the remaining infectivity was stable for over 7 hours. This non-specific loss of infectivity has been previously described ^17^ and may relate to the formation of aggregates, i.e., reduction in the number of particles, rather than proteolytic degradation.

The effect of high concentrations of reducing agents on prion infectivity is difficult to predict, with the role of the disulphide bridge on prion propagation being uncertain.^23-26^ We find that even high concentrations of DDT (e.g. 30 mM) have no influence on infectivity when homogenates are treated prior to infection. Interestingly, when we assessed the effects of applying DTT to the cells during the exposure to RML prions, even low concentrations of DTT (i.e. 1 mM), that had no significant effect on cell viability, rendered the cells resistant to RML prion infection, with saturation occurring at 5 mM before cell toxicity was observed at concentrations of 10 mM and greater. The contribution of the native disulphide to the stability of PrP^C^ has been studied with several logs molar excess of DTT required for reduction.^27,28^ Therefore as these concentrations DTT would not reduce the disulphide bond in PrP^C^, it is likely that inactivation of another cellular protein by disulphide reduction is responsible for the inhibition of infectivity as has been suggested by the inhibition of cell-free PrP conversion assays.^23,26^

The protein transfection agent, Pro-Ject, a cationic lipid ^29^ is used to deliver proteins into cells in an active form and is likely to act by coating the protein in a positively charged layer of amphipathic molecules so that it is readily taken up by the negatively charged surface of the cytoplasmic membrane. In agreement with this, we find that treatment of RML prions with Pro-Ject was found to enhance infectivity by almost an order of magnitude. In contrast, treatment with anionic lipids led to a reduction in apparent infectivity, demonstrating that the polarity may be important to the process.

Finally, we measured the contact time and prion concentration necessary for the cell to become infected. This is informative, not only from the point of view of experimental design, but also because kinetic investigations of this type provide a useful way of probing mechanisms. It is noteworthy that the dependence of cells infected on the exposure time follows an exponential time-course, implying a single rate-limiting process. The transmission half-time was dependent on prion concentration and found to be about 20 min at saturating RML concentrations. This shows that transmission is coupled to a process occurring over this time scale and is not instantaneously achieved by a rapid tight-binding event, i.e., any initial binding event can be reversed. Our kinetic data are consistent with an initial relatively weak binding event with an apparent dissociation constant of around 700 infectious units ml^−1^ at the cell surface. This process is followed by an entry mechanism that has a half time of about 20 min.

## MATERIALS AND METHODS

### Preparation of Prion-Infected Brain Homogenates

All procedures were carried out in a microbiological containment level III facility with strict adherence to safety protocols. Care of mice was performed according to institutional and national animal care committee guidelines. As described previously,^17^ brains from 200 terminal CD-1 mice infected with the RML prion strain ^14^ were prepared as 10 % (w/v) homogenates in Dulbecco's phosphate buffered saline lacking Ca^2+^ or Mg^2+^ ions (D-PBS) (Invitrogen, UK) using tissue grinders and pooled to produce a large stock of 10% (w/v) RML brain homogenate (designated I6200). This homogenate was used whole without clarification and was briefly re-homogenized by vortexing before use. One batch of 18 brains from uninfected CD-1 mice were homogenized to produce a pool of 10% (w/v) normal CD-1 brain homogenate. RML brain homogenate (I6200) [10% (w/v)] was titred in CD1 mice and the infectious prion titer was 1 × 10^8.3^ intracerebral LD_50_ / ml.^17^

### Treatment of RML Brain Homogenates

#### Freeze/Thawing

A 1 ml aliquot of RML brain homogenate (I6200) [10% (w/v)] was snap-frozen in liquid nitrogen for 5 min. The sample was then left to thaw at room temperature. A 100 μl aliquot was removed. This was regarded as one freeze/thawing cycle. The remaining RML brain homogenate (I6200) was snap-frozen in liquid nitrogen for 5 min and thawed at room temperature a further 15 times. After 5, 10 and 15 cycles, 100 ml aliquots were removed. All samples were serially diluted into Optimem supplemented with foetal calf serum (OFCS) from 3 × 10^−5^ and 3 × 10^−7^ and examined in the ASCA.

#### Incubation at Different Temperatures

RML brain homogenate (I6200) [10% (w/v)] in D-PBS was incubated in microtubes without agitation at 4, 20 or 37°C for a 7 hour period. The samples were incubated in the presence or absence of 1 x complete protease inhibitors (Roche Applied Science, UK). 100 μl aliquots of each homogenate were removed at 1 hour intervals and frozen at − 80°C. The aliquots were diluted 1 × 10^−5^ and 3 × 10^−6^ into cell culture medium (OFCS, Opti-MEM, containing 10% foetal calf serum (FCS, Invitrogen, UK), 100 U/ml penicillin and 100 μg/ml streptomycin (Invitrogen, UK) and examined using the ASCA and compared to same aliquot of directly frozen homogenate.

#### Centrifugation

A 500 ml aliquot of RML brain homogenate (I6200) [10% (w/v)] was centrifuged in a microfuge (Eppendorf, 5415D) at 13,200 rpm for 10 min. The supernatant was isolated and the pellet was resuspended in D-PBS to the original starting volume. Aliquots of non-fractionated RML brain homogenate (I6200), supernatant and resuspended pellet fractions were assayed in the ASCA using 5 dilutions between 3 × 10^−5^ and 3 × 10^−7^ into OFCS.

#### Reducing Agents (Dithiothreitol, DTT)

RML brain homogenate (I6200) [10% (w/v)] was incubated for 1 hour at room temperature with 0, 1, 5, 10, 20, and 30 mM DTT (Sigma, UK) respectively. The homogenates were then serially diluted into OFCS (with concomitant dilution of DTT) and assayed by ASCA. To determine the effect of DTT on cell susceptibility 0, 1, 5, 10, 20 and 30 mM DTT were directly added to the culture medium (OFCS) during the ASCA infections of serially diluted RML homogenates (I6200). Cell viability was determined by trypan blue staining (see below).

#### Lipids

The following lipid mixes were prepared: an anionic mix (10 mg phosphatidyl choline and 10 mg phosphatidyl glycerol; Lipid products, UK), a neutral mix (16 mg phosphatidyl choline and 4 mg phosphatidyl ethanolamine, Lipid products, UK) and a cationic mix (20 mg Pro-Ject, Pierce, UK). A blank microtube was used as a control. All mixtures were vacuum-dried overnight and separately added to a solution of RML brain homogenate (I6200) [10% (w/v)] diluted one hundred-fold in PBS. This was followed by 5 min incubation at room temperature and 2 min of agitation using a vortex. Samples were serially diluted into OFCS and analyzed using the ASCA.

### Cultures of Prion-Susceptible PK-1 Cells

PK1 cells, a line that is highly susceptible to RML prions and derived from N2a cells ^15^ were routinely grown into OFCS medium using 15cm Petri dishes.

### Automated Scrapie Cell Assay

The ASCA was performed with an adapted automated protocol.^15^ The infection steps and cell splits were carried out on an automation platform (Biomeck FX) from Beckman Coulter. 18000 PK-1 cells per well in 200 μl OFCS were inoculated into a 96-well plate and kept at 37°C in a 5% CO_2_ incubator. After 16 hours the medium was replaced with 300 μl of sample diluted in OFCS. A serially diluted sample of RML brain homogenate (I6200) [10% (w/v)] was run in parallel with the unknown samples for titer quantification. After 1, 2, 4 and 8 h or 3 days incubation at 37°C the cells were split 1:8 and transferred to a new 96-well plate containing OFCS. The cells were passaged 1:8 every three days. After the third passage an aliquot of 25,000 cells was seeded onto a 96-well Elispot plate (Millipore, UK). The plates were vacuum drained and dried at 50°C. Unless otherwise stated, the next steps were carried out at room temperature and all solutions were filtered. To each well of the 96-well PVDF plate 60 μl of 1 μg/ ml proteinase K (PK) (Roche, UK) in lysis buffer (50 mM Tris HCl (Sigma, UK) pH 8; 150 mM NaCl (Sigma, UK); 0.5% Na deoxycholate (Sigma, UK); 0.5% Triton-X 100 (Sigma, UK) were added. This was followed by 30 min incubation at 37°C. The plates were washed twice with 160 μl PBS by suction, incubated for 10 min with 120 ml of 2 mM PMSF (Sigma, UK). Next, 120 μl of a 3 M guanidinium thiocyanate (>99%, Sigma, UK) solution in 10 mM Tris HCl (Sigma, UK) pH 8.0 were added for 20 min. The plates were washed seven times with 160 μl PBS. Afterwards 120 μl of Superblock dry blend (TBS) blocking buffer (Perbio, UK) was added to each well and incubated for 1 hour followed. The plates were exposed to 0.6 μg/ml anti-PrP ICSM18 (D-Gen Ltd, London; 1:5,000) prepared in TBST (10 mM Tris HCl,150 mM NaCl, 0.1% Tween 20, pH 8) containing 1 % non-fat dry milk for 1 hour. The plates were washed five times with 160 ml TBST, incubated for 1 hour with 60 μl goat anti-mouse alkaline phosphatase-conjugated anti-IgG1 (Southern Biotechnology Associates; US) 1:7000 in TBST-1% non-fat dry milk and washed again five times with 160 μl TBST. Plates were incubated for 16 min with 54 μl AP dye (BIO-RAD, US). The plates were washed twice with water, dried and stored at −20°C. The number of PrP^Sc^-positive cells was determined with a Zeiss KS Elispot system (Stemi 2000-C stereo microscope equipped with a Hitachi HV-C20A color camera, a KL 1500 LCD scanner and Wellscan software from Imaging Associates, Oxfordshire, UK).

### Trypan Blue Staining and Cell Counting

A 96-well hydrophobic protein binding PVDF Elispot plate (Millipore, UK) was activated by adding 50 μl ethanol (Fisher, UK) to each well followed by drawing off and rinsing with 120μl PBS. Next, an aliquot of cells (<1000) was seeded onto the plate, filtered through the membrane and left to dry at 50°C for 2h. Trypan blue (Sigma, UK) was diluted 1:10 in lysis buffer. 100 μl of trypan blue solution was then added to each well of the dried 96-well plate, left for 1 minute and then drawn off. The cell number was determined using the Elispot imaging system as described above.

### Quantitative Analyses

PrP^Sc^ positive spots were converted into Tissue Culture Infectious Units (TCIU). The mean spot number obtained from a serial dilution of the titred RML brain homogenate (I6200) with known intra-cerebral LD_50_ units/ml were fitted into the following equation:

$${\text{Spot count=LD}}_{\text{50}}{}^{\text{n}}{\text{.max//(LD}}_{\text{50}}{}^{\text{n}}{\text{+ K}}^{\text{n}}\text{) }$$

Values for max, K and n were derived from this fit. Max = maximum spot count; K= half maximal, n = cooperativity.

To calculate TCIU from spot count for unknown samples we used the relationship:

TCIU=exp(ln F//n)

where F = spot count.K^n^/(max-spot count).

Statistical analyses were performed using two-tailed unpaired t-tests as reported in the figure legends.

## ABBREVIATIONS

RML: Rocky Mountain Laboratories
SCA: scrapie cell assay
ASCA: automated scrapie cell assay
PrP: prion protein
CJD: Creutzfeldt-Jakob disease
BSE: bovine spongiform encephalopathy
PrP^C^: normal cellular prion protein
PrP^Sc^: scrapie isoform of prion protein
TCIU: tissue culture infectious unit
DTT: dithiothreitol
OFCS: Optimem supplemented with foetal calf serum
D-PBS: Dulbecco's phosphate buffered saline lacking Ca^2+^ or Mg^2+^ ions
FCS: foetal calf serum
PK: proteinase K
PMSF: phenylmethylsuphonyl fluoride
AP: alkaline phosphatase
